# Modulation of Leukocyte Behavior by an Inflamed Extracellular Matrix

**DOI:** 10.1155/2000/51902

**Published:** 2000

**Authors:** Hagai Schor, Gayle G. Vaday, Ofer Lider

**Affiliations:** The Department of Immunology The Weizmann Institute of Science Rehovot Israel

**Keywords:** Adhesion, Leukocytes, Proteinase, Heparanase, Migration

## Abstract

Inflammation is a response of the immune system to foreign insult or physical damage. Various cellular and humoral components of the immune system are recruited from the vascular system and are translocated through endothelium, and into extracellular matrix (ECM) compartments of inflamed tissues. This translocation is orchestrated by various types of accessory signals, in the form of soluble or complexed molecules, which evoke remarkable transitions in leukocyte activities. Recruited inflammatory cells give rise to mechanisms of migration, including the secretion of enzymes and other pro-inflammatory mediators and the alteration of their adhesive contacts with the ECM. Hence, migrating cells secrete enzymes, chemokines, and cytokines which interact with the ECM, and thereby, provide the cells with intrinsic signals for coordinating their responses. Resultant products of enzymatic modifications to the ECM microenvironment, such as cytokine- and ECM-derived molecules, may be also part of a cell-signaling mechanism that provides leukocytes with information about the nature of their inflammatory activity; such a mechanism may give the immune system data that can be cognitively interpreted for consequential activities. This article reviews the findings that support this notion and describe the dynamic interactions between participants of the inflammatory processes.


*Developmental Immunology*, 2000, Vol. 7(2-4), pp. 227-238
Reprints available directly from the publisher
Photocopying permitted by license only

© 2000 OPA (Overseas Publishers Association)
N.V. Published by license under the
Harwood Academic Publishers imprint,
part of the Gordon and Breach Publishing Group.
Printed in Malaysia

### Modulation of Leukocyte Behavior by an Inflamed Extracellular Matrix

HAGAI SCHOR, GAYLE G. VADAY and OFER LIDER*

*The Department of Immunology, The Weizmann Institute of Science, Rehovot, Israel*

Inflammation is a response of the immune system to foreign insult or physical damage. Various cellular and humoral components of the immune system are recruited from the vascular system and are translocated through endothelium, and into extracellular matrix (ECM) compartments of inflamed tissues. This translocation is orchestrated by various types of accessory signals, in the form of soluble or complexed molecules, which evoke remarkable transitions in leukocyte activities. Recruited inflammatory cells give rise to mechanisms of migration, including the secretion of enzymes and other pro-inflammatory mediators and the alteration of their adhesive contacts with the ECM. Hence, migrating cells secrete enzymes, chemokines, and cytokines which interact with the ECM, and thereby, provide the cells with intrinsic signals for coordinating their responses. Resultant products of enzymatic modifications to the ECM microenvironment, such as cytokine- and ECM-derived molecules, may be also part of a cell-signaling mechanism that provides leukocytes with information about the nature of their inflammatory activity; such a mechanism may give the immune system data that can be cognitively interpreted for consequential activities. This article reviews the findings that support this notion and describe the dynamic interactions between participants of the inflammatory processes.

*Keywords:* Adhesion, Leukocytes, Proteinase, Heparanase, Migration

*Abbreviations:*ECM, extracellular matrix, HS, heparan sulfate, MMP, matrix metalloproteinase, MIP-1β, macrophage inflammatory protein 1β, RANTES, regulated upon activation normal T cell expressed and secreted, TIMP, tissue inhibitor of metalloproteinases, TNF, tumor necrosis factor, uPA, urokinase plasminogen activator, uPAR, urokinase plasminogen activator receptor

### INTRODUCTION

Inflammation is a highly coordinated, localized immune response to injury or a foreign particle introduced into the body. Most forms of inflammation are amplified, accelerated and propagated by the recruitment of humoral and cellular components of the immune system, especially the site-specific accumulation and subsequent activation of leukocytes. Circulating leukocytes are "patrolling" mobile units of the immune system which facilitate a rapid and efficient response to tissue insult. While naive lymphocytes normally migrate into secondary lymphoid tissues where antigens are presented to invoke differentiation, mature memory and effector lymphocytes distinctly home to certain sites in the likelihood of

* Address for correspondence: Dr. Ofer Lider, The Department of Immunology, The Weizmann Institute of Science, Rehovot, Israel 76100. Fax: 972–8–934–3657; E-mail: ofer.lider@weizmann.ac.il encountering their specific antigen or context for effector function. During inflammation, mature lymphocytes, primarily memory T cells, extravasate through blood vessel walls, and migrate through extracellular matrix (ECM) towards sites of inflammation. These processes require coordination of a vast array of cellular and molecular mechanisms to efficiently abate injury, resolve inflammation, and sustain homeostasis (Springer, 1994; Butcher and Picker, 1996; Adams and Shaw, 1994).

The ECM, which is made of a tissue-specific variable mixtures of different types of proteoglycans, glycoproteins, and glycosaminoglycans, has a structural and mechanical role in supporting and circumscribing tissue compartments. It serves as a secondary barrier to the passage of fluids and disseminating cells into the extravascular compartment (Shimizu and Shaw, 1991). It is becoming increasingly clear that in addition to its structural importance, the ECM provides resident or patrolling cells with immunological information by sending regulatory messages that promote cell proliferation, differentiation, activation, and migration. Thus, the ECM serves as a specialized reservoir of regulatory factors, which may include macromolecules (i.e., collagen type IV, fibronectin, laminin, heparan sulfate [HS] proteoglycans), proteases and their inhibitors, and cytokines. Changes in the composition of these factors greatly influence the outcome of the inflammatory response (Shimizu and Shaw, 1991; Nathan and Sporn, 1991), as the inflammatory microenvironment changes dynamically from a "latent" form to a modified, "activated" form thereby promoting inflammatory cell responses. This transition provides particular subtypes of leukocytes with contextual information to signal and foster the appropriate cellular responses, including cell activation, adhesion, and migration (Gilat et al, 1996). Activated leukocytes and other cells that constitute the dynamic microenvironment are also capable of affecting their surroundings. These cells release a wide range of cytokines, chemokines, growth factors, and other inflammatory mediators, together with matrix-degrading enzymes, into the inflammatory milieu (Madri et al, 1996; Owen and Campbell, 1999). These molecules and their derivatives presented in the context of the ECM may act reciprocally to either promote or diminish inflammatory processes. Recent findings and perspectives of these concepts are presented in this review.

### LYMPHOCYTE INTERACTIONS WITH THE ECM

Lymphocytes recruited into inflamed sites have sensory mechanisms that enable them to process and cognitively interpret the input data from the "activated" ECM and to correctly resolve the output responses. These cellular sensory systems are dependent on membrane receptors that interact with adhesive ligands and mediate cell-cell and cell-matrix interactions. Lymphocyte adhesion to ECM is enhanced upon cell activation and is crucial not only for extravasation and migration into inflammation sites, but also adhesion is linked to the release of cytokines, growth factors and matrix-degrading enzymes. Lymphocytes stimulation by cytokines, chemokines, or chemical agents, such as phorbol esters, leads to conformational changes in cell surface expressed, ECM-specific $\beta_1$ integrins from low to high affinity ligand binding states. Thus, integrin-mediated adhesion and de-adhesion resulting from these changes facilitate cell migration (Hynes, 1992; Shattil and Ginsberg, 1997; Clark and Brugge, 1995).

The major transducers of signals from the extracellular milieu into lymphocytes are the $\beta_1$ integrin family of membrane receptors, which is comprised of a common $\beta$ chain (CD29) paired with one of several $\alpha$ chains. The engagement of integrins with their ligands lead to tyrosine phosphorylation and integrin-cytoskeleton interactions, resulting in expression of several genes, such as pro-inflammatory cytokines and chemokines (Shimizu and Shaw, 1991). Leukocytes and other cells migrating into the inflammatory milieu, including fibroblasts, keratinocytes, and epithelial cells, may secrete soluble mediators that bind the ECM. ECM-anchored mediators include cytokines and chemokines, such as TNF$\alpha$ (Alon et al, 1994; Hershkoviz et al, 1995), IFN$\gamma$ (Lortat et al, 1991; Fernandez-Botran et al, 1999), IL-7 (Ariel et al,

1997), IL-2 (Ariel et al, 1998), and RANTES and MIP-1β (Gilat et al, 1994), as well as the growth factors bFGF (Yayon et al, 1991; Bashkin et al, 1989) and TGFβ (Yamaguchi et al, 1990). Thus, ECM and inflammatory mediators may act concomitantly to transmit synergistic signals to immune cells, such as lymphocytes, and thereby regulate and restrict immune reactions to inflammatory loci.

### BIOLOGICAL RAMIFICATIONS OF CYTOKINE INTERACTIONS WITH THE ECM

Cytokines sequestered at sites of inflammation may be anchored to ECM constituents, thus serving as a storage depot that affects newly recruited, proximal leukocytes. A chemoattractant gradient may also be created in this context, further attracting and activating incoming leukocytes. In addition, ECM-cytokine interactions may modulate leukocyte activity to restricted sites of inflammation. For example, TNFα which is a key mediator of immune responses, binds avidly to the major ECM constituents fibronectin (Alon et al, 1994) and laminin (Hershkoviz et al, 1995). TNFα binding to fibronectin is mediated by its 30-kDa amino-terminal domain, at a site distinct from the heparin- and fibrin-binding domains (Alon et al, 1994). This TNFα-fibronectin association augments $\beta_1$ integrin-dependent adhesion and increases cellular protein tyrosine phosphorylation of $CD4^+$ T cells (Hershkoviz et al, 1994). Moreover, activated lymphocytes adhere to fibronectin- or laminin-TNFα substrates more avidly than cells exposed to soluble TNFα and immobilized fibronectin or laminin substrates (Hershkoviz et al, 1995; Hershkoviz et al, 1994).

Chemokines are low molecular weight molecules which have important roles in many processes, such as hematopoiesis, tumor immunology, angiogenesis, HIV infection, and lymphocyte trafficking (Rollins, 1997; Baggiolini et al, 1997; Ben-Baruch et al, 1995). Chemokine interactions with cell surface- or ECM-associated glycosaminoglycans such as HS (Tanaka et al, 1993; Witt and Lander, 1994), are important for directing leukocyte migration. Chemokines bind to their G-coupled receptors on leukocytes and activate a cascade of intracellular signals, including augmentation of $\beta_1$and $\beta_2$ integrin-ligand affinities (Lloyd et al, 1996; Carr et al, 1996). Thus, ECM-bound chemokines and cytokines amplify the adhesion of T cells to activated endothelium and ECM (Taub et al, 1993). Moreover, ECM-bound chemokines affect the preferential migration of neutrophils and T cells by creating haptotactic gradients of chemoattractants (Taub et al, 1993; Webb et al, 1993; Franitza et al, 1999).

The ECM serves as a scaffold for dynamic processes, such as cell migration and locomotion, and the diffusion and binding of factors. The kinetic properties of these processes were recently analyzed using T cells migrating in a three-dimensional ECM-like gel in real-time (Franitza et al, 1999). IL-2 conditioned T cells embedded in IL-2 and RANTES gradients formed in ECM gels polarized immediately after encountering the chemoattractants. Subsequently, fractions of these cells migrated, in either a random or directional fashion, towards the chemoattractant sources, while other cells remained polarized but stationary. The number of T cells migrating directionally towards RANTES or IL-2 (which served as key representative molecules for chemokines and cytokines, respectively) peaked in concordance with the formation of chemotactic gradients. Thus, the kinetics of gradient-formation affect the migration-related activities and kinetics of migration. T cell responses to the matrix-associated chemical gradients probably involve the modulation of both the composition and the function of cytoskeletal and signal transducing elements. Such elements can include GTPases, GTPase exchange factors, $p125^{FAK}$, and actin-binding proteins (Ratner et al, 1997). IL-2R and pertussis toxin-sensitive receptors mediated the directional migration of T cells towards IL-2 and RANTES respectively. The directional and, to a lesser degree, the random locomotion of T cells induced by both chemoattractants required intact tyrosine kinase signaling (Franitza et al, 1999). Moreover, preferential VLA-4 and VLA-5 interactions with fibronectin contributed to the directional migration of T cells, while VLA-6-laminin and VLA-2-collagen interactions inactive at the physiological pH of 7.2 (Gilat et al, 1995). Thus, heparanase activity is restricted to anatomical sites harboring acidic conditions, such as sites of inflammation and tumor growth. Interestingly, heparanase binds to ECM at physiological pH, and may promote $\beta_1$-integrin-mediated adhesion of $CD4^+$ T cells to ECM. These findings are supported by evidence of increased mobility of IL-2 activated T cells at pH 6.7 compared to pH 7.1 in ECM gels (Ratner, 1992b), suggesting that increased migration through ECM may correlate with enhanced activity of heparanase or other degradative enzymes. Moreover, heparanase-binding to ECM may function similar to cytokines or chemokines bound to ECM, which facilitate the recruitment of cells to inflamed loci. As inflammatory stimuli arise and the physical condition of the surroundings change, heparanase exerts its enzymatic activity.

Supporting this notion was the recent findings which delineated the enzymatic potential of connective tissue-activating peptide-III (CTAP-III) (Hoogewerf et al, 1995; Rechter et al, 1999). CTAP-III is a member of the C-X-C chemokine subfamily (Lida, 1996). CTAP-III has been shown to have various activities, such as induction of plasminogen activator activity (Castor et al, 1990) and stimulation of histamine release from basophils (Baeza et al, 1990). CTAP-III has also been detected in wound fluid (Matsuoka and Grotendorst, 1989) and the sera of rheumatoid arthritis patients (Castor et al, 1993), therefore CTAP-III may serve as mediator of inflammation. In addition to these characteristics, CTAP-III derived from platelets (Hoogewerf et al, 1995), as well as T cells and neutrophils (Rechter et al, 1999), exhibits heparanase activity at slightly acidic pH conditions, similar to the lower-pH-dependency for placental heparanase activity (Gilat et al, 1995). Together, these findings support the idea that CTAP-III may function as at least one of the heparanases that facilitate immune cell migration into ECM during inflammation. Enzymes, such as CTAP-III, that are released by immune cells in the context of ECM may serve as utility factors with multiple capacities to modify the environment and modulate inflammatory-cell behavior.

The enzymatic cleavage of the ECM probably creates a path through the matrix for leukocyte migration. During this process, resultant degradation products are released into the inflammation milieu. Recently, we have found that heparanase degradation of HS moieties in the ECM produces molecules that can affect inflammatory processes. Such ECM-derived products were found to be tri-sulfated disaccharides. Derivatives of heparin, a molecule chemically-related to HS glycosaminoglycans, produced by the action of heparinase yields disaccharides with activities similar to disaccharides derived from ECM. These disaccharides were shown to suppress *in vivo* T cell-mediated delayed-type hypersensitivity reaction (Lider et al, 1995) and arrested the progression of adjuvant arthritis in rats (Cahalon et al, 1997). Moreover, these disaccharides were shown to reduce the activity of TNFα secreted from various *in vitro* stimulated immune cells, such as T cells, macrophages, and mast cells (Lider et al, 1995; Cahalon et al, 1997).

Since the disaccharides were derived from the ECM environment through which immune cells adhere and migrate, the disaccharides' effects on T cell adhesion to ECM substrates were analyzed. Treatment of T cells with heparan sulfate and heparin derived disaccharides stimulated T cell adhesion to endothelial cells, ECM, and fibronectin (unpublished observations). The adhesion induced by disaccharides involves specific integrins of the $\beta_1$ sub-family and is targeted to the RGD sequence on FN. Moreover, the disaccharide-induced adhesion of T cells involves T cell-expressed G protein-coupled receptors and a cascade of events in which the signaling of protein kinase C plays a role. The up-regulation of T cell integrin affinities by disaccharides can be considered an active process that is mediated through diverse intracellular signaling pathways, rather than a passive interruption to T cell-substrate interactions. Interestingly, the adhesion of T cells to fibronectin was inhibited when these cells were exposed to the disaccharides, together with distinct accessory molecules in the same environment. These accessory signals were found to be chemokines such as MIP-1β and RANTES, but not other pro-adhesive stimuli,

(IL-2, PHA or CD3 cross-linking; Hershkoviz et al, 2000). Similar to their anti-adhesive effects, the disaccharides inhibited MIP-1β-induced T cell chemotaxis through FN-coated polycarbonate filter membranes in trans-well migration assays. Thus, ECM-derived disaccharides appear to inhibit T cell adhesion and migration induced by chemokines, since they did not inhibit T cell integrin-mediated adhesion induced by non-chemokine activators.

The mechanisms by which heparin- and HS-derived disaccharides exert their adhesion- and chemotactic migration-modifying capacities are not yet fully understood. However, the biological functions of the enzyme-generated disaccharide molecules were not restricted to their induction of activation of T cell adhesion. Disaccharide compounds inhibited T cell migration specifically induced by chemokines. It has been reported that down-regulation of the function of chemokine receptors on leukocytes may result from de-sensitization of chemokine-specific receptors occurring from changes in phosphorylation of the intracellular domain of the receptor (Ben-Baruch et al, 1997). These findings support a mechanism by which disaccharide molecules can exert their inhibition of chemokine-mediated processes via certain active mechanisms, namely, the desensitization of specific chemokine receptors. Such moieties of heparin and HS proteoglycans, its related molecules, or other ECM byproducts generated by enzymatic action may inherently affect cell behavior by exerting antiinflammatory properties, representing yet another fundamental loop in the regulation of inflammatory events.

### B. Urokinase plasminogen activator

Plasminogen activator (PA) and its specific inhibitors (PAIs) have a central role in the enzymatic cascades which govern matrix production, remodeling, and turnover. These enzymatic cascades involve interactions of PA, MMPs and other serine proteinases that may yield activation of one another's zymogens (Blasi, 1997; Vassalli et al, 1991). Active uPA derived from pro-uPA, binds to its membrane receptor uPA-receptor (uPAR) and stimulates the proteolytic activity. The proteolysis is modulated by the binding of PAIs to this complex. The active uPA preferentially cleaves plasminogen-zymogen and activates the generation of plasmin. Plasmin, a serine protease, is significant for fibrinolysis and ECM renovation processes. The ECM interactions with components of this cascade emphasize its importance in inflammation. Associations of PA and PAI with heparin (Andrade-Gordon et al, 1986) and PAI- and uPAR-vitronectin interaction (Blasi, 1997) have been described. These interactions highlight the importance of ECM components in promoting activation and de-activation of uPA-mediated proteolysis by sequestering these molecules and providing substrates for cell adhesion. Upon dissociation of PAI from the uPA-uPAR-complex, uPA-mediated cleavage takes place, and plasminogen, which is found in fibrin clots or bound to its cell surface receptor, is converted to active plasmin (Vassalli et al, 1991). The interaction of the cascade components with cell and ECM substrates in the vicinity of the enzymatic action restricts and localizes the process, and thus enables proper migration toward the inflamed site. Moreover, uPA-uPAR binding can induce exposure of a chemotactic epitope and stimulate chemotaxis of activated leukocytes (Fazioli, 1997; Hoyer-Hansen, 1997). Thus, uPA binding to uPAR can affect local proteolytic or chemotactic capabilities of cells within the inflammatory milieu.

Several lines of evidence have clarified the significance of the uPA system in T lymphocyte functions during inflammation. Binding of uPA-uPAR facilitates invasion into a fibrin matrix by Jurkat T lymphoma cells (Kramer et al, 1994) and activation of peripheral blood T lymphocytes both *in vivo* and *in vitro* (Nykjaer et al, 1996). In addition, PMA stimulated HUT 78 lymphoid cells secrete a modulator that induces uPA activity (Osada et al, 1996). T cells, derived from an HIV-positive donor, express high levels of uPAR. Yet, healthy donor T cell-expression is amplified by *in vitro* stimulation with phorbol ester, mitogens, cytokines (IL-2, IL-4, IL-7) and upon co-clustering of the TCR complex and integrins via treatment with specific antibodies (Bianchi et al, 1996). Subsequently, plasminogen activation by these cells augments their invasion in an ECM gel (Nykjaer et al, 1994). Degradation of tenascin C, an anti-adhesive ECM protein, by plasmin converts the ECM protein from anti- to pro-adhesive for lymphocytes. Thus, ECM cleavage by migrating cells may yield potent factors that affect cell behavior and cell-matrix interactions.

### C. Matrix metalloproteinases (MMPs) interactions with the ECM

Migrating leukocytes express MMPs to facilitate movement across tissue barriers. Leukocytes rely on these enzymes to mediate their extravasation and penetration into tissues during inflammation (Owen and Campbell, 1999; Goetzl et al, 1966; Woessner, 1991). The MMP family of enzymes shares a common structural domain and can collectively cleave every ECM substrate (Woessner, 1991). MMP zymogens are activated by the action of plasmin or other enzymatic or chemical mechanisms, as well as by other activated MMPs, by exposing an intrinsic zinc ion that is essential for their activity. MMP activities are restrained and regulated by various inhibitors, particularly the family of endogenous tissue inhibitors of metalloproteinases (TIMPs). TIMPs exert their activity by binding non-covalently to MMPs (Goetzl et al, 1996), and have been detected as soluble inhibitors in the body fluid (TIMP-1and TIMP-2), or anchored to the ECM (TIMP-3). The high abundance of TIMPs in both body fluids and ECM environment emphasizes the necessity to modulate MMPs activity. In addition, MMP expression and secretion is tightly regulated at the level of gene transcription (Mauviel, 1993). MMPs involvement has been implicated in normal tissue remodeling during development, menstruation, wound repair following tissue injury, as well as in pathological states, such as metastatic cancer, multiple sclerosis, rheumatoid arthritis, and periodontal disease(Goetzl et al, 1996; Woessner, 1991; Owen and Campbell, 1999).

In general, MMPs are synthesized *de novo* and are rapidly secreted by migrating leukocytes after stimulation with specific pro-inflammatory inducers, such as IL-1, TNFα, PDGF, and prostaglandin E2 (Mauviel, 1993; Johnatty et al, 1997; Leppert et al, 1995). In addition, chemokines, such as RANTES, MIP-1α and MIP-1β induce the expression of MMP-9 (Johnatty et al, 1997). T cell activation by IL-2 greatly augments MMP-9 and MMP-2 expression, the predominant MMPs of T cells, and thereby, migration across gels of basement membrane (Leppert et al, 1995). In contrast, interferon-γ, progesterone, and corticosteroids are among the inflammatory mediators which may suppress MMP synthesis (Woessner, 1991; Johnatty et la, 1997). Interestingly, several of the cytokines and chemokines that modulate the expression of MMPs and induce adhesion and migration of T cells, are anchored to ECM moieties. Thus, these mediators may have a profound impact on cell migration since they affect both cell adhesion and influence MMPs synthesis. Moreover, adhesion of immune cells by itself can induce VCAM-1 mediated expression of 72-kDa gelatinase (Romanic and Madri, 1994), and cell-matrix interactions via $\beta_1$ integrins control MMP-dependent migration through basement membrane (Xia et la, 1996). Therefore, MMPs expression is regulated by several mechanisms that modulate their activity during cell extravasation from the blood and migration through inflamed tissues.

MMP interactions with and disruption of ECM proteins may result in the release of growth factors and inflammatory mediators bound and sequestered in the context of migration through matrix (Imai et al, 1997). The same cytokines that can induce metalloproteinase production may also be substrates for cleavage by MMPs. For example, MMPs are capable of processing and inactivating the proinflammatory cytokine IL-1β (Ito et al, 1996), while the release of membrane-bound TNFα (McGeehan et al, 1994) augments and sustains inflammation. Therefore, MMPs may either promote the activity of pro-inflammatory signals or reduce their impact. Together, these findings emphasize the important role of MMPs in regulating inflammatory responses by not only clearing a path for migrating cells, but also modulating the contextual signals that affect immune cell activity.

### D. Elastase, more than a ECM degrading enzyme

Human leukocyte elastase (HLE) is a serine proteinase that degrades various matrix substrates, such as elastin, collagen, fibronectin, and HS proteoglycans. HLE and its catalytic activity depend on an intrinsic serine residue in its active site. Various cell types secrete elastase, including macrophages, neutrophils, and T cells (Bieth, 1986). Elastase has been shown to be important in several biological process, such as induction of cytokine secretion (Bedard et al, 1993), cell activation (Renesto and Ghignard, 1993), and degradation of clotting factors (Taylor et al, 1977). Moreover, elastase can degrade and release surface molecules, such as CD2, CD4, and CD8, from T cell surface and therefore, affect their physiology in inflammatory diseases (Doring et al 1995). Binding of elastase to neutrophils and monocytes can regulate the avidity and pro-adhesiveness of the integrin MAC-1 (CD11b/CD18) (Cai and Wright 1996).

Elastase is a potent enzyme that possesses immense proteolytic and destructive potential. It is stored in its active form within leukocyte granules (Gallin, 1984). Therefore, there is a necessity to restrict and tightly regulate elastase activity. Indeed, this necessity is fulfilled by natural inhibitors of serine proteinases, termed serpins, which actually comprise about 10% of total plasma proteins. Upon cellular activation by TNFα and IL-8, HLE is translocated to the plasma membrane. This mechanism ensures a focused localization of the enzymes' biological activities within specific proximity (Owen et al, 1997). Both soluble and membrane-bound forms of elastase are synthesized by T cells (Bristow et al, 1991). HLE is predominantly synthesized by neutrophils, yet, T cell elastase, in moderate levels, can make contributions in modifying cell-matrix interactions, ECM moieties, and cytokine activities as needed during inflammatory episodes. Moreover, the release of elastase from T cells in moderation may actually be a mode of preventing dysregulated proteolysis.

In addition its ECM degrading activity, elastase may be involve in the cleavage of inflammatory cytokines and their receptors (Porteu et al, 1991), and therefore modify their action. The elastase-mediated cytokine-modification may enhance cytokine activity, as was shown for IL-8 and IL-1 (Black et al, 1991; Padrines et al, 1994), or may inactivate it (Scuderi et al, 1991). A recent study in our laboratory suggested that elastase cleaves IL-2, a pro-inflammatory cytokine and chemoattractant, to produce peptides with a regulatory functions. These IL-2 peptides have been shown to inhibit T-cell migration through FN-coated membranes induced by IL-2 and MIP-1β as well as to inhibit T-cell adhesion to FN induced by IL-2, IL-7, MIP-1β, PMA, anti-CD3, and anti-$\beta_1$-integrin-activating mAb. Thus, the enzymatic products of IL-2 may serve as natural inhibitors of inflammation (Ariel et al, 1998). Consequently, HLE may coordinate the performance of inflammatory mediators, such as cytokines, by generating either active or inactive forms to contribute in regulating the inflammatory response.

### ECM DEGRADATION YIELDS A NEW CONTEXT WITH NOVEL SIGNALS

Immune cells adapt their behavior according to contextual information introduced to them by the constantly changing environment of the ECM. Thus, alteration of ECM consonance may transduce novel regulatory signals that are important for cell adaptation to a new environment (Lukashev and Werb, 1998). During inflammation, there are changes in the equilibrium between ECM constituents, such as FN, collagen, and HS proteoglycans, as well as introduction of new adhesion molecules which are not usually abundant in ECM (Clark, 1996; Guadiz et al, 1997). ECM degradation products may also have regulatory properties. For example, chemotactic FN fragments may selectively attract monocytes and other leukocytes into inflamed tissue (McDonald and Kelley, 1980; Norris et al, 1982). Fragments of laminin (Tanaka et al, 1997), collagen type IV (Nakagawa et al, 1999), and fibrin (Gray et al, 1995) also exhibit regulatory roles in immune cell migration and proliferation during inflammatory processes. The notion that tissue-degrading enzymes secreted by leukocytes and other cells in the inflammatory milieu can modify immune cell functions by altering the ECM context and producing small signalling molecules further highlight the importance of matrix-degrading enzymes in inflammatory processes.

### CONCLUDING REMARKS

The inflammatory microenvironment continuously and dynamically changes, enabling extravasating leukocytes to communicate with their surroundings, produce signals, and function immunologically. The ECM can be considered a physical barrier, and it has a multifactorial regulatory role in the inflammatory process. Specifically, the ECM provides cells with immunological information by sending regulatory messages that enable them to constantly adapt and fine-tune their responses and activities, in accordance with their varied surroundings (Loike et al, 1995). Moreover, the ECM can make contact with the migrating cells in its "activated" form, which facilitates proinflammatory processes, such as cell migration and recruitment. However, activated leukocytes and other cells that constitute the dynamic microenvironment are also capable of affecting their surroundings. These cells release a wide range of cytokines, chemokines, growth factors, and other inflammatory mediators, together with matrix-degrading enzymes, into the inflammatory milieu. As a consequence, immune cells are able to modulate their own environment. The modulated ECM begins to function in regulatory feedback activities upon the initial immune reaction. We propose, from the line of evidence we have discussed in this review, that immune cells have sensory mechanisms that enable them to process and cognitively interpret the input data from the modified environments, then correctly resolve the output responses. These cellular sensory systems utilize membrane receptors, including chemokine, cytokine, and adhesion receptors, which are molecules that participate in intercellular and cell-tissue interactions. The real-time sensing and processing ability of the immune system enables it to respond to a specific antigen and "cognitively" modify its activity in response to changing contextual information.

### *References*

Adams D.H. and Shaw S. (1994). Leucocyte-endothelial interactions and regulation of leucocyte migration. Lancet. 343, 831–836.

Alexander C.M. Werb Z. (1989). Proteinases and extracellular matrix remodeling. Curr. Opin. Cell Biol. 1, 974–982.

Alon R., Cahalon L., Hershkoviz R., Elbaz D., Reizis B., Wallach D., Akiyama S.K., Yamada K.M., and Lider O. (1994). TNF-$\alpha$ binds to the N-terminal domain of fibronectin and augments the $\beta$-integrin-mediated adhesion of CD4+ T lymphocytes to the glycoprotein. J. Immunol. 152, 1304–1313.

Andrade-Gordon P. and Strickland S. (1986). Interaction of heparin with plasminogen activators and plasminogen: effects on activation of plasminogen. Biochemistry. 25, 4033–4040.

Ariel A., Hershkoviz R., Cahalon L., Williams D.E., Akiyama S.K., Yamada K.M., Chen C., Alon R., Lapidot T., and Lider O. (1997). Induction of T cell adhesion to extracellular matrix or endothelial ligands by soluble or matrix-bound interleukin-7. Eur. J. Immunol. 27, 2562–2570.

Ariel A., Yavin E.J., Hershkoviz R., Avron A., Franitza S., Hardan I., Cahalon L., Fridkin M., and Lider O. (1998). IL-2 induces T cell adherence to extracellular matrix: inhibition of adherence and migration by IL-2 peptides generated by leukocyte elastase. J. Immunol. 161, 2465–2472.

Baeza M.L., Reddigari S.R., Kornfeld D., Ramani N., Smith E.M., Hossler P.A., Fischer T., Castor C.W., Gorevic P.G., and Kaplan A.P. (1990). Relationship of one form of human histamine-releasing factor to connective tissue activating peptide-III. J. Clin. Invest. 85, 1516–1521.

Baggiolini M., Dewald B., and Moser B. (1997). Human chemokines: an update. Annu. Rev. Immunol. 15, 675–705.

Bashkin P., Doctrow S., Klagsbrun M., Svahn C.M., Folkman J., and Vlodavsky I. (1989). Basic fibroblast growth factor binds to subendothelial extracellular matrix and is released by heparitinase and heparin-like molecules. Biochemistry. 28, 1737–1743.

Bashkin, P., Razin, E., Eldor, A. and Vlodavsky, I. (1990). Degranulating mast cells secrete an endoglycosidase that degrades heparan sulfate in subendothelial extracellular matrix, Blood. 75, 2204–12.

Bedard M., McClure C.D., Schiller N.L., Francoeur C., Cantin A., and Denis M. (1993). Release of interleukin-8, interleukin-6, and colony-stimulating factors by upper airway epithelial cells: implications for cystic fibrosis. Am. J. Respir. Cell Mol. Biol. 9, 455–462.

Ben-Baruch A., Michiel D.F., and Oppenheim J.J. 1995. Signals and receptors involved in recruitment of inflammatory cells. J. Biol. Chem. 270, 11703–11706.

Ben-Baruch, A., Grimm, M., Bengali, K., Evans, G. A., Chertov, O., Wang, J. M., Howard, O. M., Mukaida, N., Matsushima, K. and Oppenheim, J. J. (1997). The differential ability of IL-8 and neutrophil-activating peptide-2 to induce attenuation of chemotaxis is mediated by their divergent capabilities to phosphorylate CXCR2 (IL-8 receptor B). *J. Immunol.* 158, 5927–5933.

Bianchi E., Ferrero E., Fazioli F., Mangili F., Wang J., Bender J.R., Blasi F., and Pardi R. (1996). Integrin-dependent induction of functional urokinase receptors in primary T lymphocytes. J. Clin. Invest. 90, 1133–1141.

Bieth J.G. 1986. Elastases: catalytic and biological properties. In *Biology of Extracellular Matrix: Regulation of Matrix Accumulation* (Mecham R.P., ed.), Academic Press, Orlando, Florida 217–320.

Black R., Kronheim S., Sleath P., Greenstreet T., Virca G.D., March C., and Kupper T. (1991). The proteolytic activation of interleukin-1 beta. Agents Actions Suppl. 35, 85–89.

Blasi F. (1997). uPA, uPAR, PAI-1: key intersection of proteolytic, adhesive, and chemotactic highways? Immunol. Today. 18, 415–417.

Bristow C.L., Lyford L.K., Stevens D.P., and Flood P.M. (1991). Elastase is a constituent product of T cells. Biochem. Biophys. Res. Comm. 181, 232–239.

Butcher E.C. and Picker L.J. (1996). Lymphocyte homing and homeostasis. Science. 272, 60–66.

Cahalon L., Lider O., Schor H., Avron A., Gilat D., Hershkoviz R., Margalit R., Eshel A., Shoseyev O., and Cohen I.R. (1997). Heparin disaccharides inhibt tumor necrosis factor α production by macrophages and arrest immune inflammation in rodents. Intl. Immunol. 9, 1517–1522.

Cai T.Q.. and Wright S.D. (1996). Human leukocyte elastase is an endogenous ligand for the integrin CR3 (CD11b/CD18, Mac-1, $\alpha_M\beta_2$) and modulates polymorphonuclear leukocyte adhesion. J. Exp. Med. 84, 1213–1223.

Carr M.W., Alon R., and Springer T.A. (1996). The C-C chemokine MCP-1 differentially modulates the avidity of β1 and β2 integrins on T lymphocytes. Immunity. 4, 179–187.

Castor C.W., Andrews P.C., Swartz R.D., Ellis S.G., Hossler P.A., Clark M.R., Matteson E.L., and Sachter E.F. (1993). The origin, variety, distribution, and biologic fate of connective tissue activating peptide-III isoforms: characteristics in patients with rheumatic, renal, and arterial disease. Arthritis Rheum. 36, 1142–1153.

Castor C.W., Walz D.A., Johnson P.H., Hossler P.A., Smith E.M., Bignall M.C., Aaron B.P., Underhill P., Lazar J.M., Hudson D.H., Cole L.A., Perini F., and Mountjoy K. (1990). Connective tissue activation. XXXIV: Effects of proteolytic processing on the biologic activities of CTAP-III. J. Lab. Clin. Med. 116, 516–526.

Clark E.A. and Brugge J.S. (1995). Integrins and signal transduction pathways: the road taken. Science. 268, 233–239.

Clark R.A.F. (1996). Wound Repair. In *The Molecular and Cellular Biology of Wound Repair* (Clark R.A.F., ed.) Plenum, New York 1–50.

Doring G., Frank F., Boudier C., Herbert S., Fleischer B., and Bellon G. (1995). Cleavage of lymphocyte surface antigens CD2, CD4, and CD8 by polymorphonuclear leukocyte elastase and cathepsin G in patients with cystic fibrosis. J. Immunol. 154, 4842–4850.

Fazioli F., Resnati M., Sidenius N, Higashimoto Y., Appella E., and Blasi F. (1997). A urokinase-sensitive region of the human urokinase receptor is responsible for its chemotactic activity. EMBO J. 16, 7279–7286.

Fernandez-Botran R., Yan J., Justus DE. (1999). Binding of interferon gamma by glycosaminoglycans: a strategy for localization and/or inhibition of its activity. Cytokine. 11, 313–325.

Folkman J, Klagsbrun M, Sasse J, Wadzinski M, Ingber D, Vlodavsky I. (1988). A heparin-binding angiogenic protein-basic fibroblast growth factor is stored within basement membrane. Am J Pathol, 130, 393–400.

Franitza S., Alon R., and Lider O. (1999). Real-time analysis of integrin-mediated chemotactic migration of T lymphocytes within 3-D extracellular matrix gels. J. Immunol. Methods. 225, 9–25.

Fridman, R., Lider, O., Naparstek, Y., Fuks, Z., Vlodavsky, I. and Cohen, I. R. (1987). Soluble antigen induces T lymphocytes to secrete an endoglycosidase that degrades the heparan sulfate moiety of subendothelial extracellular matrix, J Cell Physiol, 130, 85–92.

Gallin J.I. (1984). Neutrophil specific granules: a fuse that ignites the inflammatory response. Clin. Res. 32, 320–328.

Gilat D., Cahalon L., Hershokoviz R., and Lider O. (1996). Interplay of T cells and cytokines in the context of enzymatically-modified extracellular matrix. Immunol. Today. 17, 16–20.

Gilat D., Hershkoviz R., Goldkorn I., Cahalon L., Korner G., Vlodavsky I., and Lider O. (1995). Molecular behavior adapts to context: heparanase functions as an extracellular matrix-degrading enzyme or as a T cell adhesion molecule, depending on the local pH. J. Exp. Med. 181, 1929–1934.

Gilat D., Hershkoviz R., Mekori Y.A., Vlodavsky I., and Lider O. (1994). Regulation of adhesion of CD4+ T lymphocytes to intact or heparinase-treated subendothelial extracellular matrix by diffusible or anchored RANTES and MIP-1β. J. Immunol. 153, 4899–4906.

Goetzl E.J., Banda M.J., and Leppert D. (1996). Matrix metalloproteinases in immunity. J. Immunol. 156, 1–4.

Gray A.J., Bishop J.E., Reeves J.T., Mecham R.P., and Laurent G.J. (1995). Partially degraded fibrin(ogen) stimulates fibroblast proliferation in vitro. Am. J. Respir. Cell Mol. Biol. 12, 684–690.

Guadiz G., Sporn L.A., and Simpson-Haidaris P.J. (1997). Thrombin cleavage-independent deposition of fibrinogen in extracellular matrices. Blood. 90, 2644–2653.

Gunderson D., Tran-Thang C., Sordat B., Mourali F., and Ruegg C. (1997). Plasmin-induced proteolysis of tenascin-C. J. Immunol. 158, 1051–1060.

Hershkoviz R, Schor H, Ariel A, Hecht I, Cohen I.R, Lider O, Cahalon L. (2000). Disaccharides Generated from Heparan Sulfate or Heparin Modulate Chemokine-Induced T Cell Adhesion to Extracellular Matrix Immunology. 99, 87-93.

Hershkoviz R., Cahalon L., Miron S., Alon R., Sapir T., Akiyama S.K., Yamada K.M., and Lider O. (1994). TNF-α associated with fibronectin enhances phorbol myristate acetate- or antigen -mediated integrin-dependent adhesion of CD4+ T cells via protein tyrosine phosphorylation. J. Immunol. 153, 554–565.

Hershkoviz R., Goldkorn I., and Lider O. (1995). Tumour necrosis factor-a interacts with laminin and functions as a pro-adhesive cytokine. Immunology. 85, 125–130.

Hoogewerf A.J., Leone J.W., Reardon I.M., Howe W.J., Asa D., Heinrikson R.L. and Ledbetter S.R. (1995). CXC chemokines connective tissue activating peptide-III and neutrophil activating peptide-2 are heparin/heparan sulfate-degrading enzymes. J. Biol. Chem. 270, 3268–3277.

Hoyer-Hansen G., Ploug M., Behrendt N., Ronne E., and Dano K. (1997). Cell-surface acceleration of urokinase-catalyzed receptor cleavage.. Eur J. Biochem. 243, 21–26.

Hynes R.O. (1992). Integrins: versatility, modulation, and signaling in cell adhesion. Cell. 69, 11–25.

Ilda N., Haisa M., Igarashi A., Pencev D., and Grotendorst G.R. (1996). Leukocyte-derived growth factor links the PDGF and CXC chemokine families of peptides. FASEB J. 10, 1336–1345.

Imai K., Hiramatsu A., Fukushima D., Pierschbacher M.D., and Okada Y. (1997). Degradation of decorin by matrix metalloproteinases: identification of the cleavage sites, kinetic analyses and transforming growth factor-beta1 release. Biochem. J. 322, 809–814.

Ito A., Mukaiyama A., Itoh Y., Nagase H., Thogersen I.B., Enghild J.J., Sasaguri Y., and Mori Y. (1996). Degradation of interleukin 1beta by matrix metalloproteinases. J. Biol. Chem. 271, 14657–14660.

Johnatty R.N., Taub D.D., Reeder S.P., Turcovski-Corrales S.M., Cottam D.W., Stephenson T.J., and Rees R.C. (1997). Cytokine and chemokine regulation of proMMP-9 and TIMP-1 production by human peripheral blood lymphocytes. J. Immunol. 158, 2327–2333.

Kjellen L. and Lindahl U. (1991). Proteoglycans: structures and interactions. Annu. Rev. Biochem. 60, 443–475.

Korner G., Bjornsson T., and Vlodavsky I. (1993). Extracellular matrix produced by cultured corneal and aortic endothelial cells contains active tissue-type and urokinase-type plasminogen activators. J. Cell. Physiol. 154, 456–463.

Kramer M.D., Spring H., Todd R.F., and Vettel U. (1994). Urokinase-type plasminogen activator enhances invasion of human T cells (Jurkat) into a fibrin matrix. J. Leukocyte Biol. 56, 110–116.

Leppert D., Hauser S.L., Kishiyama J.L., An S., Zeng L., and Goetzl E.J. (1995). Stimulation of matrix metalloproteinase-dependent migration of T cells by eicosanoids. FASEB J. 9, 1473–1481.

Leppert D., Waubant E., Galardy R., Bunnett N.W., and Hauser S.L. (1995). T cell gelatinase mediate basement membrane transmigration in vitro. J. Immunol. 154, 4379–4389.

Lider O., Cahalon L., Gilat D., Hershkoviz R., Siegel D., Margalit R., Shoseyev O., and Cohen I.R. (1995). A disaccharide that inhibits tumor necrosis factor a is formed from the extracellular matrix by the enzyme heparanase. Proc. Natl. Acad. Sci. USA. 92, 5037–5041.

Lloyd A.R., Oppenheim J.J., Kelvin D.J., and Taub D.D. (1996). Chemokines regulate T cell adherence to recombinant adhesion molecules and extracellular matrix proteins. J. Immunol. 156, 932–938.

Loike J.D., El Koury J., Cao L., Richards C.P., Rascoff H., Mandeville J.T.H., Maxfield F.R., and Silverstein S.C. (1995). Fibrin regulates neutrophil migration in response to interleukin 8, leukotriene B4, tumor necrosis factor, and formyl-methionyl-leucyl-phenylalanine. J. Exp. Med. 181, 1763–1772.

Lortat, J. H., Kleinman, H. K. and Grimaud, J. A. (1991). High-affinity binding of interferongamma to a basement membrane complex (matrigel). J. Clin. Invest. 87, 878–883.

Lukashev M.E. and Werb Z. (1998). ECM signalling: orchestrating cell behaviour and misbehaviour. Trends Cell Biol. 8, 437–441.

Madri J.A., Graesser D., and Haas. T. (1996). The roles of adhesion molecules and proteinases in lymphocyte transendothelial migration. Biochem. Cell Biol. 74, 749–757.

Matsuoka J. and Grotendorst G.R. (1989). Two peptides related to platelet-derived growth factor are present in human wound fluid. Proc. Natl. Acad. Sci. USA. 86, 4416–4420.

Mauviel A. (1993). Cytokine regulation of metalloproteinase gene expression. J. Cell. Biochem. 53, 288–295.

McDonald J.A. and Kelley D.G. (1980). Degradation of fibronectin by human leukocyte elastase. Release of biologically active fragments. J. Biol. Chem. 255, 8848–8858.

McGeehan G.M., Becherer J.D., Bast R.C., Boyer C.M., Champion B., Connolly K.M., Conway J.G., Furdon P., Karp S., Kidao S., McElroy A.B., J. Nichols, Pryzwansky K.M., Schoenen F., Sekut L., Truesdale A., Verghese M., Warner J., and Ways J.P. (1994). Regulation of tumour necrosis factor-α processing by a metalloproteinase inhibitor. Nature. 370, 558–561.

Montgomery A.M.P., Sabzevari H., and Reisfeld R.A. (1993). Production and regulation of gelatinase B by human T-cells. Biochim. Biophys. Acta. 1176, 265–268.

Nakagawa H., Takano K., and Kuzumaki H. (1999). A 16-kDa fragment of collagen type XIV is a novel neutrophil chemotactic factor purified from rat granulation tissue. Biochem. Biophys. Res. Commun. 256, 642–645.

Nathan C. and Sporn M. (1991). Cytokines in context. J. Cell Biol. 113, 981–986.

Norris D.A., Clark R.A., Swigart L.M., Huff J.C., Weston W.L., and Howell S.E. (1982). Fibronectin fragments are chemotactic for human peripheral blood monocytes. J. Immunol. 129, 1612–1618.

Nykjaer A., Moller B., Todd R.F., Christensen T., Andreasen P.A., Gliemann J., and Petersen C.M. (1994). Urokinase receptor: an activation receptor in human T lymphocytes. J. Immunol. 152, 505–516.

Osada H., Kono T., Miwa K., and Yamada C. (1996). Phorbol-ester-stimulated human lymphoid cell lines produce a plsminogen activator modulator inducing cell-bound urokinase-type plasminogen activator in malignant tumor cell lines. Int. J. Cancer. 65, 178–185.

Owen C.A. and Campbell E.J. (1999). The cell biology of leukocyte-mediated proteolysis. J. Leukocyte Biol. 65, 137–150.

Owen C.A., Campbell M.A., Boukedes S.S., and Campbell E.J. (1997). Cytokines regulate membrane-bound leukocyte elastase on neutrophils: a novel mechanism for effector activity. Am. J. Physiol. 272, L385–393.

Padrines M., Wolf M., Walz A., and Baggiolini M. (1994). Interleukin-8 processing by neutrophil elastase, cathepsin G and proteinase-3. FEBS Lett. 352, 231–235.

Porteu F., Brockhaus M., Wallach D., Engelmann H., and Nathan C.F. (1991). Human neutrophil elastase releases a ligand-binding fragment from the 75-kDa tumor necrosis factor (TNF) receptor. Comparison with the proteolytic activity responsible for shedding of TNF receptors from stimulated neutrophils. J. Biol. Chem. 5, 18846–18853.

Ratner S. (1992b). Motility of IL-2-stimulated lymphocytes in neutral and acidified extracellular matrix. Cell. Immunol. 139, 399–410.

Ratner, S. (1992a) Lymphocyte migration through extracellular matrix, Invasion Metastasis, 12, 82–100.

Ratner, S., Sherrod, W. S., Lichlyter, D. (1997). Microtubule retraction into the uropod and its role in T cell polarization and motility. J. Immunol. 159, 1063.

Rechter M., Lider O., Cahalon L., Baharav E., Dekel M., Seigel D., Vlodavsky I., Aingorn H., Cohen I.R., and Shoseyov O. (1999). A cellulose-binding domain-fused recombinant human T cell connective tissue-activating peptide-III manifests heparanase activity. Biochem. Biophys. Res. Comm. 255, 657–662.

Renesto P. and Ghignard M. (1993). Enchancement of cathepsin G-induced platelet activation by leukocyte elastase: consequence for the neutrophil-mediated platelet activation. Blood. 82,139–144.

Rollins B.J. (1997). Chemokines. Blood. 90, 909–928.

Romanic A.M. and Madri J.A. (1994). The induction of 72-kD gelatinase in T cells upon adhesion to endothelial cells is VACM-1 dependent. J. Cell Biol. 125, 1165–1178.

Scuderi P., Nez P.A., Duerr M.L., Wong B.J., and Valdez C.M. (1991). Cathepsin-G and leukocyte elastase inactivate human tumor necrosis factor and lymphotoxin. Cell. Immunol. 135, 299–313.

Shattil S. and Ginsberg M.H. (1997). Integrin signalling in vascular biology. J. Clin. Invest. 100, 1–5.

Shimizu Y. and Shaw S. (1991). Lymphocyte interactions with extracellular matrix. FASEB J. 5, 2292–2299.

Springer T.A. (1994). Traffic signals for lymphocyte recirculation and leukocyte emigration: the multistep paradigm. Cell. 76, 301–314.

Tanaka Y., Adams D.H., Hubscher S., Hirano H., Siebenlist U., and Shaw S. (1993). T-cell adhesion induced by proteoglycan-immobilized cytokine MIP-1β. Nature. 361, 79–82.

Taub D.D., Conlon K., Lloyd A.R.., Oppenheim J.J., and Kelvin K.J. (1993). Preferential migration of activated CD4+ and CD8+ T cells in response to MIP-1α and MIP-1β. Science. 260, 355–358.

Taylor J.C., Crawford I.P., and Hugli T.E. (1977). Limited degradation of third component (C3) of human complement by human leukocyte elastase (HLE): Partial characterization of C3 fragments. Biochemistry. 16, 3390–3396.

Turnbull, J. E., Fernig, D. G., Ke, Y., Wilkinson, M. C. and Gallagher, J. T. (1992). Identification of the basic fibroblast growth factor binding sequence in fibroblast heparan sulfate, J. Biol. Chem, 267, 10337–10341.

Vassalli J.-D., Sappino A.-P., and Belin D. (1991). The plasminogen activator/plasmin system. J. Clin. Invest. 88,1067–1072.

Vlodavsky, I., Eldor, A., Haimovitz, F. A., Matzner, Y., Ishai, M. R., Lider, O., Naparstek, Y., Cohen, I. R. and Fuks, Z. (1992). Expression of heparanase by platelets and circulating cells of the immune system: possible involvement in diapedesis and extravasation, Invasion Metastasis, 12, 112–127.

Walker, A., Turnbull, J. E. and Gallagher, J. T. (1994) Specific heparan sulfate saccharides mediate the activity of basic fibroblast growth factor, *J. Biol. Chem*, 269, 931–935.

Webb L.M.C., Ehrengruber M.U., Clark-Lewis I., Baggiolini M., and Rot A. (1993). Binding to heparan sulfate or heparin enhances neutrophil responses to interleukin 8. Proc. Natl. Acad. Sci. USA. 90, 7158–7162.

Witt D.P. and Lander A.D. (1994). Differential binding of chemokines to glycosaminoglycan subpopulations. Curr. Biol. 4, 394–400.

Woessner J. F. (1991). Matrix metalloproteinases and their inhibitors in connective tissue remodeling. FASEB J. 5, 2145–2154.

Xia M., Sreedharan S.P., Dazin P., Damsky C.H., and Goetzl E.J. (1996). Integrin-dependent role of human T cell matrix metalloproteinase activity in chemotaxis through a model basement membrane. J. Cell. Biochem. 61, 452–458.

Yamaguchi Y., Mann D.M., and Ruoslahti E. (1990). Negative regulation of transforming growth factor-b by the proteoglycan decorin. Nature. 346, 281–284.

Yanaka K., Camarata P.J., Spellman S.R., Skubitz A.P., Furcht L.T., Low W.C. (1997). Laminin peptide ameliorates brain injury by inhibiting leukocyte accumulation in a rat model of transient focal cerebral ischemia. J. Cereb Blood Flow Metab. 17, 605–611.

Yayon A., Klagsbrun M., Esko J.D., Leder P., and Ornitz D.M. (1991). Cell surface, heparin-like molecules are required for binding of basic fibroblast growth factor to its high affinity receptor. Cell. 64, 841–848.

Zhou H., Bernhard E.J., Fox F.E., and Billings P.C. (1993). Induction of metalloproteinase activity in human T-lymphocytes. Biochim. Biophys. Acta. 1177, 174–178.

